# In the Nepalese context, can a husband’s attendance during childbirth help his wife feel more in control of labour?

**DOI:** 10.1186/1471-2393-12-49

**Published:** 2012-06-14

**Authors:** Sabitri Sapkota, Toshio Kobayashi, Masayuki Kakehashi, Gehanath Baral, Istuko Yoshida

**Affiliations:** 1 Department of Health Promotion and Development, Graduate School of Health Sciences, Hiroshima University, Hiroshima, Japan; 2 Department of Health Informatics, Graduate School of Health Sciences, Hiroshima University, Hiroshima Japan; 3 Paropakar Maternity and Women’s Hospital, Kathmandu, Nepal; 4 Department of Nursing Science, Faculty of Health Care, Tenri Health Care University, Nara, Japan

## Abstract

**Background:**

A husband’s support during childbirth is vital to a parturient woman’s emotional well-being. Evidence suggests that this type of support enables a woman to feel more in control during labour by reducing maternal anxiety during childbirth. However, in Nepal, where childbearing is considered an essential element of a marital relationship, the husband’s role in this process has not been explored. Therefore, we examined whether a woman in Nepal feels more in control during labour when her husband is present, compared to when another woman accompanies her or when she has no support person.

**Methods:**

The study participants were low risk primigravida women in the following categories: women who gave birth with their husband present (n = 97), with a female friend present (n = 96), with mixed support (n = 11), and finally, a control group (n = 105). The study was conducted in the public maternity hospital in Kathmandu in 2011. The Labour Agentry Scale (LAS) was used to measure the extent to which women felt in control during labour. The study outcome was compared using an F-test from a one-way analysis of variance, and multiple regression analyses.

**Results:**

The women who gave birth with their husband’s support reported higher mean LAS scores (47.92 ± 6.95) than the women who gave birth with a female friend’s support (39.91 ± 8.27) and the women in the control group (36.68 ± 8.31). The extent to which the women felt in control during labour was found to be positively associated with having their husband’s company during childbirth (β = 0.54; p < 0.001) even after adjusting for background variables. In addition, having a female friend’s company during childbirth was related to the women’s feeling of being in control during labour (β = 0.19; p < 0.001) but the effect size was smaller than for a husband’s company.

**Conclusion:**

The results show that when a woman’s husband is present at the birth, she feels more in control during labour. This finding has strong implications for maternity practices in Nepal, where maternity wards rarely encourage a woman to bring her husband to a pregnancy appointment and to be present during childbirth.

## Background

The extent to which a woman feels in control during labour is viewed as central to maternal emotional well-being in childbirth. According to Hodnett and Simmons-Tropea [1], control (a woman’s sense of mastery over internal and environmental factors) during childbirth has far-reaching consequences for the emotional well-being of a new mother. Research has shown that when women in labour have a greater sense of control, this helps to reduce maternal anxiety during childbirth [2,3] and ultimately leads to a more positive birth experience [4]. Although the meaning of control during labour varies between women [5,6], labour support from a birth companion has been found beneficial in reducing maternal distress, and is a major step towards greater personal control [3,5,7,8]. Campero *et al.*[4] present an anecdotal record of how birth companions help women to feel in control during labour. On the other hand, the same anecdotal record notes that for women who are alone, labour is a long and exhausting effort, full of uncertainties, and with no indication of how soon the baby will be born. A number of randomised, controlled trials [8] have demonstrated the impact a birth companion can have, and the World Health Organization (WHO) has recommended that a parturient woman be allowed to have a birth companion she trusts and with whom she feels at ease [9,10]. However, these recommendations do not tend to be followed in facility-based births in many developing countries, including Nepal.

The presence of a birth companion is clearly linked both to positive birth outcomes and a greater degree of personal control during labour [8,11] in Western society. However, there is no clear understanding of whether a husband’s attendance during childbirth in developing countries enables his wife to feel more in control of the labour process. This is because feeling in control is a subjective experience, and is affected by the cultural norms and practices of the society [12,13]. In addition, with younger women, who were giving birth for the first time, their greater perception of labour pain and antenatal worries related to labour were found to be associated with a lower feeling of being in control during labour [5]. Most importantly, past studies on support during labour in industrialised countries have shown that the presence of professional doulas, midwives and a partner/husband during labour helped the women achieve greater control during labour. However, these support people are different from those commonly available and used in developing countries [8,11], where lay (untrained) female company (an in-law or a neighbour/friend) provides the main support [9]. This is why, despite evidence-based practice, many maternity health care practitioners question whether a parturient woman in developing countries can benefit from her husband’s company during childbirth in the same way as women in industrialised countries [14].

Nevertheless, in a limited resource setting there is an argument that continuous support for a woman in labour, either from her husband or from a female friend, could have a significant impact on her emotional well-being [8] . This is because, in the limited resource setting of developing countries, women are more prone to feeling lonely in a birthing environment, and this leads to increased maternal distress during childbirth [15]. Therefore, in developing countries, where gender disparities and discrimination are highly prevalent, the impact of the husband’s active involvement on maternal emotional well-being during pregnancy and childbirth needs to be explored. Without exploring this, the comprehensive role of the husband in maternity care cannot be clearly understood. There is therefore a need for cross-cultural studies in developing countries such as Nepal, to evaluate continuous labour support and its impact on the emotional well-being of the mother. This type of study is also essential if there is to be a more active role for husbands during childbirth in a patriarchal society like Nepal, where the husband’s knowledge about pregnancy and childbirth heavily influences whether his wife seeks maternity care [16]. Therefore, this quasi-experimental study was carried out in Nepal to examine whether a husband’s attendance during childbirth has an impact on his wife’s sense of personal control during labour. The experience of wives whose husbands were present during childbirth was compared with that of women supported by a female friend, and women who had no support at all. The hypothesis was that the husband’s company during childbirth would increase the woman’s sense of control during labour, compared to the women with other birth partners (female friend) or those with no support.

### Husband’s role during childbirth in Nepal

A partner’s support during childbirth is vital to the emotional well-being of parturient women. In Nepal, however, husbands are completely prohibited from entering delivery rooms. Although husbands are allowed to make occasional visits to their wife inside the labour room, they are generally to be found outside the labour and delivery rooms looking after the supplies of medication, food and drinks. They are only contacted by the nurses and physicians in emergencies. The husband’s presence inside the delivery room is culturally discouraged because there is a belief that his presence will make labour pain worse and prolong the labour [17,18]. Traditionally, therefore, women have been assisted during childbirth by a lay female friend, one of their in-laws or a neighbour [19]. On the other hand, the availability of female company in urban society has been challenged by growing urbanisation, internal migration, busy lifestyles and nuclear families [16,20,21]. As a result, husbands who used to feel shy about accompanying their pregnant wife in public have gradually begun to attend antenatal clinic with her. Likewise, husbands who preferred their mother or sister-in-law to take care of everything to do with the birth of their child are now taking the initiative and going to the health care facilities when their wife gives birth [17]. These changes in urban society clearly indicate that husbands have become more interested in issues connected with pregnancy and childbirth in recent years. In such a changing context, tertiary level hospitals in Nepal are allowing husbands to support their wife during labour in the birthing centre. However, in a culture where this is discouraged and where women have low social status, it is still unclear whether the husband’s presence in the delivery room has a positive impact on his wife’s feeling of being in control during labour.

Previous studies in Nepal have explored the experiences of husbands and wives separately following the birth of a baby where the husband was present at the birth. The husbands described their experiences positively, but confessed that they had overwhelmingly emotional feelings [18]. Wives, on the other hand, reported that emotional support from their husbands outweighed their emotional discomfort, including their own hesitation and their concern for their husband [22]. Nevertheless, to make the practice more evidence-based and culturally acceptable, it is necessary to explore whether a woman in Nepal feels more in control during labour and childbirth when her husband is present, compared to when another woman accompanies her or when she is left completely alone.

The data for this study were obtained from part of a broader investigation which examined the impact of the husband’s presence on maternal emotional well-being during childbirth and postpartum in Nepal.

## Methods

### Setting

This study was carried out in the Paropakar Maternity and Women’s Hospital (PMWH), located in Kathmandu, Nepal. This is a central level referral hospital for obstetrics and gynaecology, with 22,000 deliveries per year [23]. The hospital was chosen deliberately because women are allowed to be accompanied by their husband or another person during labour and childbirth inside its birthing unit.

### Participants and eligibility criteria

The participants were primigravida women who were admitted to give birth to their babies between February and April 2011. In total, 2766 primigravida women were admitted to the hospital during the study period. After screening approximately one third of these women (admitted between 8:00 and 18.00 each day), 314 women who met the inclusion and exclusion criteria were all approached and asked to participate. Initially, all these women voluntarily consented to take part in the study. However, five of them declined to be interviewed after the birth of the baby without giving a reason for their withdrawal. This left a total of 309 women (accompanied only by their husband (n = 97), accompanied only by a female friend (n = 96), with both their husband and a female friend (n = 11), and the controls (n = 105)) who completed this study.

The women were assessed for their eligibility to participate in the study using pre-defined criteria. Only primigravida women (aged between 18 and 35 years) with singleton pregnancy at full term gestation (37 to 42 weeks), and no history of obstetric, medical or psychological problems were asked to participate. Only women residing within 90 min of the hospital by public transportation were recruited, as the broader study also comprised the follow-up criteria. Women were considered eligible to participate in this study if their husband or a female friend was available to stay in the hospital throughout the birth process. However, women were excluded from the study if they had chosen to have a caesarean section, had cervical dilatation of more than four centimetres at the time of admission, or were having induction and augmentation.

### Recruiting procedures

The women were screened for inclusion and exclusion criteria at the time of admission, after they had been examined by the attending doctor. Recruitment was carried out between 8:00 and 18.00 every day within the study period. All the women who met the eligibility criteria were approached regarding their participation in the study. This way, each day, on average one to two women were enrolled in each group. Their confidential, written, informed consent was obtained while they were in the latent phase of labour.

### The women in the support group

**Figure 1:**
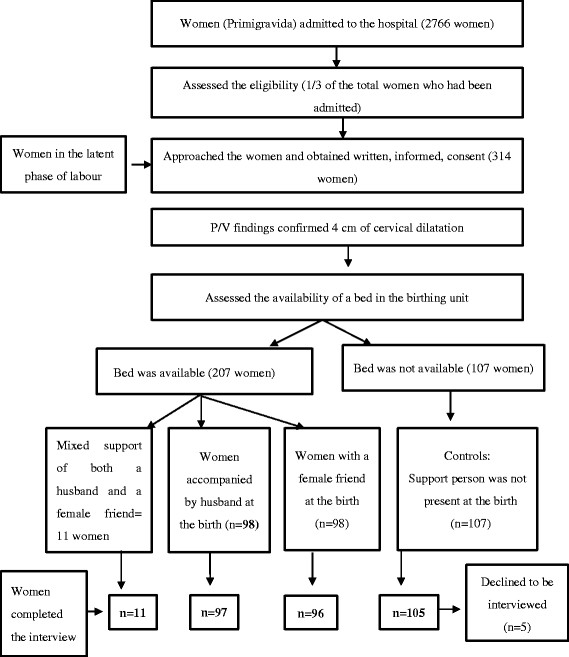
Flow diagram of the enrolment procedure for women in the support and control groups from the time of admission to the hospital through the completion of the interview.

The allocation of the women to the support and control groups was based on the availability of beds in the birthing unit at the time the per-vaginal examination confirmed four centimetres of cervical dilatation. A birth companion (either the woman’s husband or a female friend) was assigned according to each woman’s preference. Women received continuous labour support from their birth partner until the end of the first stage, and continued to receive help throughout the second and third stages of labour. The allocation of the women to support and control groups is illustrated in Figure 1.

### The women in the control group (without a support person)

If no bed was available in the birthing unit of the hospital when per-vaginal examination revealed four centimetres of cervical dilation, the women were allotted to a control group. The women in the control group were first taken to the antenatal care unit until the cervix was fully dilated (10 centimetres), and thereafter they were transferred to the delivery room for the actual birth of the baby. Women in the control group had also had their support person (either their husband or a female friend) present during the first stage of labour, but they had not been with them constantly. Most importantly, the support person was not present while these women were giving birth to the baby.

### Instruments

#### Socio-demographic data questionnaire

Background questionnaires were developed and administered to collect the socio-demographic and antenatal characteristics of the women and their husbands.

#### Recording sheet

A recording sheet was developed to record the length (in minutes) and type of labour (induced/spontaneous), the mode of delivery (vaginal/instrumental/caesarean), the use of syntocinon (yes/no), the presence of complications (yes/no) and details of the new-born baby. This information was primarily collected by reviewing the medical records of each woman after the birth of the baby.

#### Labour agentry scale (LAS)

To measure the main study outcome, the Labour Agentry Scale (LAS), originally developed in English by Hodnett and Simmons-Tropea [1], was used in this study. The 10-item LAS includes six positive and four negative descriptions of the extent to which women feel in control during childbirth. Women rank the items on a 7-point scale from (1) ‘almost all of the time’ to (7) ‘never, or almost never’. A high score denotes greater control, and a low score minimal control. For the purposes of this study, the original scale was translated into Nepali after obtaining permission (both for translation and use) from the original author (Hodnett, E.D.). The scale was translated by two independent translators, and discrepancies in terms of wording were settled in discussion with maternity health care experts. The translated scale was pre-tested with 10 new mothers in the postnatal ward of the same hospital before the start of this study. The internal consistency of the items within the Nepali version of the LAS, estimated in this study using Cronbach’s alpha, was 0.70.

#### Visual analogue scale (VAS)

This numeric rating scale, between 0 and 10 on a 10 cm long horizontal line, was used to measure the women’s recollections of their perception of labour pain. Women were asked to rate the worst pain they experienced during the childbirth process.

### Data collection procedure

After the birth of her baby, each woman was contacted in the postnatal unit and interviewed by one of the research assistants. These research assistants had a Bachelor of Science in Nursing, and were hired from outside the hospital. They were informed about the study procedures by the first author before the study began.

Within 24 hours of the delivery, contact was made with the women who had had vaginal deliveries, in order to take account of the early discharge policy in the hospital. However, to avoid physiological discomfort to the women who had undergone a caesarean delivery, the contact period was extended to 48 hours. During contact, background information was obtained, followed by administration of the VAS and the LAS.

### Data analyses

Firstly, the distributions of all background characteristics, the birth outcomes and the perception of labour pain were compared between the three groups of women (n = 298) (women accompanied only by their husband; women accompanied only by a female friend and women in the control group), using a chi squared test for categorical variables and one-way analysis of variance (ANOVA) for continuous variables respectively. Secondly, the main outcome of the LAS score was compared across the groups of women with the three types of support, using an F-test from ANOVA, followed by a post-hoc test according to Bonferroni’s method. Multiple regression analyses were then conducted to test the study hypothesis by adjusting the background variables. To run the regression analysis, the distributions of numerical data were checked for normality using P-P charts. Variables were entered into the model by the stepwise regression method. Two dummy variables (1. The husband’s company *versus* without the husband’s company, and (2. A female friend’s company *versus* without a female friend’s company) were created for an independent variable called *type of support*. The missing values for the length of the first stage of labour (26 values) were estimated in the first round of the regression analysis. Due to the small number of women (n = 11) who had received mixed support from both their husband and a female friend during childbirth, these findings were not formally compared with the rest of the groups. However, women with mixed support were included in the regression equation in a second phase, to examine the trend of the effect of mixed support on the women’s sense of control during labour.

All the data were analysed using SPSS for Windows, version 18.0 [24], and the significance was set at alpha < 0.05 for all analyses.

## Ethical approval

The Ethical Committee of Hiroshima University in Japan and the Nepal Health Research Council approved the study.

## Results

### Women’s characteristics

The mean age of the women who participated in this study was 21.85 years (± 2.82 years), with a slightly higher mean for their husbands (25.54 years ± 4.17 years). Table 1 provides the overall background characteristics for the 309 women who participated in this study. In general, the characteristics of the women and husbands, including household and antenatal characteristics, were similar across the three groups of women. Likewise, the reported perception of labour pain was not significantly different between the three groups of women. However, in comparing the various birth outcomes, the women who were supported by their husband had a shorter first stage of labour than the women who were supported by a female friend or women in the control group. Likewise, the percentage of spontaneous labour in the control group (80.0%) was significantly lower than for the women accompanied by their husband (92.8%) or a female friend (91.7%). Therefore, these two variables were also adjusted while conducting the regression analysis. None of the women who took part in the study were aware of the option of having a birth partner (either their husband or a female friend) at the birth before the informed consent procedure. The women who declined to be interviewed (n = 5) and those with the mixed support type (n = 11) did not differ significantly in terms of their background characteristics from the rest of the groups (result not shown).

**Table 1:** Background characteristics of the women

**Characteristics**	**Overall (n = 309)**	**Women with their husband (n = 97)**	**Women with a female companion (n = 96)**	**Control (n = 105)**
Women’s characteristics				
Women’s age (years), mean (SD)	21.85 (2.82)	22.06 (3.08)	21.80 (2.56)	21.56 (2.71)
Years of marriage, mean (SD)	1.72 (1.32)	1.57 (0.91)	1.68 (1.01)	1.88 (1.81)
Women with love marriage (%)	56.6	54.6	55.2	62.9
Years of living together, mean (SD)	1.55 (1.17)	1.43 (.72)	1.46 (.78)	1.74 (1.71)
Women with more than 10 years of schooling (%)	35.5	41.2	36.5	28.6
Working women (%)	16.8	16.5	14.6	12.4
Husbands’ characteristics				
Husbands’ age (years), mean (SD)	25.54 (4.17)	26.01 (5.02)	25.27 (3.56)	25.20 (3.92)
Husbands with more than 10 years of schooling (%)	37.2	36.1	37.5	37.1
Husbands working in managerial and professional positions (%)	12.3	14.4	10.4	12.4
Household characteristics				
Hindu (%)	78.6	81.4	76.0	80.0
Brahman/Chhetri (%)	45.9	44.3	52.1	40.0
Monthly family income^a^ (Rupees), mean (SD)	13854.09 (8676.12)	14771 (8375.48)	13700 (8354.92)	13154 (9413.85)
Women in a nuclear family (%)	60.8	69.1	57.3	55.2
Support person available at home (%)	57.9	57.7	55.2	60.0
Living in own house (%)	29.1	25.8	31.3	29.5
Antenatal characteristics				
Attended antenatal clinic (at least once)	97.1	96.9	97.9	97.1
Accompanied by husband to antenatal clinic (%)	58.3	64.9	50.02	60.0
Attended birth preparation class (%)	6.1	5.2	7.3	6.7
Birth outcomes				
Length of first stage of labour, mean (SD) in minutes ^b^	706.11 (235.31)	638.03 (203.42)	738.20 (237.31)	762.30 (232.53)
Spontaneous labour (%)	88.0	92.8	91.7	80.0
Vaginal delivery (%)	83.8	84.5	85.4	81.0
Presence of intrapartum complications (%)	18.1	17.5	15.6	21.9
Percentage of male babies	59.5	64.9	58.3	55.2
Weight of the baby at birth, mean (SD) in grams	3001.63 (374.86)	3019.43 (381.51)	3004.17 (376.39)	2959.24 (357.71)
Perception of recalled labour pain VAS score, mean (SD)	8.84 (1.73)	8.83 (1.83)	8.91 (1.65)	8.78 (1.75)

^a^ Had some missing values (n = 281).

^b^Women who were given an emergency caesarean section without full dilatation of the cervix were excluded (n = 283).

There were no statistical differences between the groups at p < 0.05 except for the length of the first stage and spontaneous labour (p < 0.01).

Note: The details of the women who received mixed support, from their husband as well as a female friend (n = 11), are not shown separately in this table due to the small sample size.

### The extent to which women felt in control during labour

The women who gave birth in the presence of a support person (their husband or a female friend or both) reported higher mean LAS scores than the controls (44.04 ± 8.94 *vs.* 36.68 ± 8.31, p < 0.001). Similarly, the women who gave birth in the presence of their husbands alone reported significantly higher mean LAS scores (47.92 ± 6.95) compared to the women who gave birth in the presence of a female friend alone (39.91 ± 8.27), or the control group (36.68 ± 8.31, p < 0.001). Similarly, as shown in Table 2, women accompanied by a female friend reported a higher LAS score than the controls.

**Table 2:** Group comparisons: LAS scores (n = 298)

**Study groups**	**Study outcome**		**Post Hoc test using Bonferroni’s method**	
	**Total score of LAS Mean (SD)**	**F (df1, df2)**^**c**^	**Mean difference (95% confidence interval)**	
Women with husband (H group)	47.92 (6.95)	53.80 (2, 295)**	H vs F	8.01 (5.28, 10.74)**
Women with a female friend (F group)	39.91 (8.27)		H vs C	11.24 (8.57, 13.91)**
Women in the control group (C group)	36.68 (8.31)		F vs C	3.23 (0.55, 5.91)*

* P < 0.05, ** P < 0.001.

^c^ results from ANOVA.

### Testing the hypothesis

After adjusting for the effect of women’s perception of labour pain, their antenatal characteristics, availability of a support person at home (covariates as identified in the literature), length of first stage of labour and rate of spontaneous labour (significant background variables, Table 1), the husband’s company during childbirth (β = 0.57, p < 0.001) was more positively related to the women’s feeling of being in control during labour as compared to the women who did not have their husband’s company during childbirth. In addition to the husband’s company, a female friend’s company during childbirth was also more positively related to the women’s feeling of being in control during labour (β = 0.19, p < 0.001) than was the case for the women who did not have a female friend’s company. While comparing the effect size, as shown in Table 3, the unstandardized coefficient for the husband’s company (B = 11.22, p < 0.001) was higher than for female company during childbirth (B = 3.79, p < 0.001), confirming the study hypothesis. Among the variables adjusted in the multiple regression analysis, the VAS score (measurement of perception of labour pain), the husband accompanying the wife to the antenatal clinic, and availability of a support person at home) were also observed to be significantly associated with the women’s feeling of being in control during labour.

**Table 3:** Effect of the husband’s company during childbirth on the women’s feeling of control during labour after adjusting selected background variables (n = 298)

**Variables**	**Unstandardised coefficients**		**Standardised coefficient**	**t**
	**B**	**Std. error**	**Beta**	
Husband at childbirth	11.22	1.05	0.57	10.70**
Perception of labour pain	−1.17	0.25	−0.22	−4.71**
Female friend at childbirth	3.79	1.06	0.19	3.59**
Accompanied by husband to antenatal clinic	2.86	0.89	0.15	3.22*
Support person available at home	2.43	0.88	0.13	2.76*

^a^ R^2^ = 0 .35, F (5, 292) = 31.95, P < 0.001.

*p < 0.01, **p < 0.001.

In a second phase, we coded another dummy variable for mixed support and included this in the regression equation to examine the effect of the mixed support. The result showed that mixed support from both the husband and a female friend was positively associated with the women’s feeling of control (p < 0.001). The unstandardised coefficient observed in the model for mixed support (B = 9.55, p < 0.001) was higher than the coefficient (B = 3.84, p < 0.001) for female company alone, but was almost equal to the effect of the husband’s company alone (B = 11.22, p < 0.001).

## Discussion

As we have hypothesised, the higher mean LAS score for the women who gave birth in the presence of their husband, compared to those who were accompanied by a female friend, or those in the control group, indicates that the husband’s presence at the birth was associated with women feeling more in control during labour. Likewise, in comparison to the controls, women who gave birth with the support of a female friend had a higher LAS score. This difference was consistent even after adjusting background variables in the regression model. These findings demonstrate the importance of the presence of a support person for Nepalese women in facility-based childbirth. However, in this study, the mean LAS score of 44.04 ± 8.94 for the women with some form of support was far lower than the mean LAS score of 54.1 ± 9.7 reported for women in North American hospitals [25]. This lower LAS mean for Nepalese women can be explained by the differences in expectation and desire for control compared to the women in North America [26]. In addition, whether or not Nepalese women feel in control may be affected by the childbirth environment of the public health care facility, which is often noisy and crowded. This type of stressful environment during the hours of labour, coupled with the lack of labour support skills in the support person (the husband or a female friend) [27] may be further reasons for the lower score of LAS reported by the women in this study.

The regression analysis demonstrated that the extent to which the women felt in control during labour was positively related to the husband’s company during childbirth. This indicates that, despite traditional and culturally discouraging practices associated with the husband’s presence during childbirth [17], the women benefitted from their husband’s company. This may be due to the changing attitude of the younger generations who advocate a shared role in childbearing and childrearing, and are only minimally affected by traditional practices. The Nepalese women felt that the presence of their husband during childbirth gave them greater self-confidence and relief from emotional distress, and facilitated communication [22]. This supports the strong association demonstrated in this study between a husband’s attendance during childbirth and the extent to which his wife felt in control during labour. Alternatively, above benefit may have occurred because the women were allowed to have the support person (either the husband or a female friend) they chose. The women who had had more supportive husbands may have chosen their husband as their companion during childbirth, resulting in a greater feeling of being in control during labour than the women with a female companion during childbirth.

On the other hand, the coefficient for a female friend’s company during childbirth was smaller than for the husband’s company during childbirth, indicating that a female friend’s company was not as beneficial as the husband’s presence. These findings are expected to question the long tradition of women being supported by a female companion in Nepal. We therefore consider it important to discuss possible reasons for these findings. The female friends who accompanied women in labour in this study ranged from the woman’s mother to a neighbour. Moreover, the women were fairly young and were giving birth to their first baby within two years of their marriage. Therefore, in comparison to the emotional bond they had with their husbands, they may not have felt as comfortable with in-laws, neighbours or relatives [28], so that the presence of their husband had a greater impact. Moreover, these female friends had not acquired any labour support skills for helping a woman in labour, which means that their ability to understand the needs of these young women may have been compromised. This may be another reason why there was no additional effect of a female companion. This has important implications for programmes preparing women for giving birth. The programmes may have to evaluate whether female friends are the most appropriate main support, and whether they need to receive training in labour skills before the birth.

Among the adjusted variables in the regression equation, the husband accompanying the wife to antenatal clinic, the presence of a support person at home and perception of labour pain were also found to be related to the extent to which the women felt in control during labour. Information acquired from formal and informal antenatal classes attended by both husband and wife suggests that the husband’s presence may have helped the women to picture the childbirth environment in advance. This argument was supported by the study conducted by Mullany *et al.*[29] in Nepal, where expectant couples’ attendance at antenatal classes promoted spousal communication. This improved communication may have encouraged the couple to discuss their concerns related to childbirth, which may, in turn, have promoted a greater sense of control during labour [5]. This finding has strong implications for maternity-related practices in Nepal, where husbands are not generally welcome at their wife’s pregnancy appointment or at the birth of their child.

Moreover, having a support person available at home was positively associated with the extent to which women felt in control during labour. Some women may have found the support of a female friend who was living with them beneficial because their husband’s lack of experience of pregnancy and childbirth-related issues made them feel insecure. This is supported by the fact that some women mentioned their husband’s lack of confidence in providing physical support at the time of childbirth [22]. On the other hand, other types of support, especially from a female friend who had had experience of giving birth in the past, may have facilitated the sharing of information and fears related to childbirth issues before the birth. This may have boosted the confidence of the women, which in turn may have affected how they reported the level of control they felt during labour [5] . However, in the changing social context of Nepal, where the younger generation’s attitudes towards family, and their opportunities to interact with family and neighbours are changing [28], an additional support system is not always available to women. In other words, women need to have confidence in their husband’s ability to provide physical support during and following the birth, so husbands need to be encouraged to be involved in their wife’s pregnancy, and should be properly prepared to provide the sole support for their wives during childbirth.

As identified in the literature, perception of labour pain showed a strong negative association with the women’s sense of control in this study as well. A similar association between the perceptions of recalled labour pain and a feeling of being in control was reported by Green and Baston [5] among English women, and by Christianes *et al.*[30] among Dutch women. This shows that women’s perception of labour pain has an important impact on the extent to which they feel in control during labour, whether pharmacological methods are applied to cope with the pain (such as epidurals in Western countries) or non-pharmacological methods (such as back massaging and breathing techniques in developing countries like Nepal). Since pain and control are closely related, Hodnett *et al.*[8] emphasise that the companion should provide physical support to help the woman tolerate the intensity of the contractions, so that she can feel good about her behaviour during labour. Therefore, there should be an emphasis on the acquisition of skills, through birth preparation classes, for anyone, such as female friends and husbands, hoping to support a woman in labour.

Although this study has demonstrated the husband’s company during childbirth was associated with the extent to which women felt in control of labour in urban Nepal, there were a number of limitations to the study. Firstly, the women were not allocated randomly to the support and control groups due to logistical problems with randomisation. They were allocated instead according to the availability of beds in the birthing unit, which leaves the possibility of some subjective bias in the study. However, within the support group, the women were allowed to choose their companion. This may have increased the likelihood that highly motivated women would choose their husband, while women with less supportive husbands would choose a female companion. On the other hand, this likelihood was reduced by taking into account the background characteristics of women who chose their husband against those who chose a female friend.

Secondly, we administered the LAS retrospectively. It is possible that the women’s sense of control may have changed over time as they became more involved with the baby [31], and this may have affected the accuracy of their memory of the extent to which they were in control. However, as Hodnett and Simmons-Tropea [1] note, the LAS score remained stable, even when subjects were tested two weeks, one month and three months postpartum, suggesting that the impact of any bias was minimal. Although we used the LAS developed in the Western setting, the Nepali version of the LAS demonstrated acceptable internal consistency, with a Cronbach’s alpha of 0.70 [32]. More specifically, this study looked at feelings of control in women giving birth to their first child with the help of a skilled birth attendant in a health care facility in an urban setting. We should therefore be cautious about generalising these findings to women giving birth to a second, third or fourth child, *etc.*, or without the help of a skilled birth attendant, whether in an urban or a rural setting.

## Conclusion

In conclusion, this study reported the extent to which women feel in control during labour, which is an aspect of maternal emotional well-being that has not been a priority in facility-based childbirth in Nepal. The study demonstrated that when Nepalese husbands (as preferred by their wives) were present at the birth of their child, their wives felt more in control during labour, despite strong cultural bias against the husband’s involvement. Equally, in spite of an age-old traditional practice of having a female companion during labour, this type of support did not have the same impact as the husband’s company. These findings should intensify the debate about whether husbands in Nepal should be involved in childbirth. In the meantime, interventional studies will also be important to investigate how a companion who is well prepared (husband or female friend) impacts on the extent to which a woman feels in control during labour. Since the population of Nepal is very diverse in terms of geography, education and ethnicity, further studies on younger women’s expectations and their sense of personal control during labour are recommended.

## Competing interests

We have no competing interests to declare.

## Authors’ contribution

SS conceived the study design, coordinated recruitment of participants, trained the research assistants, performed the statistical analysis and drafted the initial manuscript. TK contributed in the design of the study, supervised the study and made critical comments in the initial draft of the manuscript. MK guided the data analysis and reviewed all drafts of the manuscript. GB helped in the design of the study, supervised during the data collection process and reviewed the manuscript. IY participated in the design of the study, contributed in statistical analysis and helped to draft the manuscript. All authors read and approved the final manuscript.

## References

[B1] Hodnett ED Simmons-Tropea DA The Labour Agentry Scale: Psychometric properties of an instrument measuring control during childbirth Res Nurs Health 1987 10 301 310 3671777 10.1002/nur.4770100503

[B2] Cheung W Ip WY Chan D Maternal anxiety and feelings of control during labor: A study of Chinese first-time pregnant women Midwifery 2007 23 123 130 17055624 10.1016/j.midw.2006.05.001 PMC7130936

[B3] Chunuan S Somsap Y Pinjaroen S Thitimapong S Nangham S Ongpalanupat F Effect of the presence of family members, during the first stage of labor, on childbirth Outcomes in a Province Hospital in Songkhla Province, Thailand Thai J Nurs Res 2009 13 1 16 27

[B4] Campero L Garcia C Diaz C Ortiz O Reynoso S Langer A “Alone, I wouldn’t have known what to do”: A qualitative study on social support during labor and delivery in Mexico Soc Sci Med 1998 47 3 395 403 9681909 10.1016/s0277-9536(98)00077-x

[B5] Green JM Baston HA Feeling in control during labour: concepts, correlates and consequences Birth 2003 30 4 235 247 14992154 10.1046/j.1523-536x.2003.00253.x

[B6] Namey EE Drapkin Lyerly A The meaning of control for childbearing women in the US Soc Sci Med 2010 71 4 769 776 20579792 10.1016/j.socscimed.2010.05.024 PMC2910238

[B7] Langer A Campero L Garcia C Reynoso S Effects of psychosocial support during labour and childbirth on breastfeeding, medical interventions, and mothers’ wellbeing in a Mexican public hospital: a randomized clinical trial Br J Obstet Gynecol 1998 105 1056 1063 10.1111/j.1471-0528.1998.tb09936.x 9800927

[B8] Hodnett ED Gates S Hofmeyr GJ Sakala C Weston J Continuous support for women during childbirth Cochrane Database Syst Rev 2011 2 CD003766 21328263 10.1002/14651858.CD003766.pub3

[B9] World Health Organization Care in normal birth: A practical guide. Maternal and newborn health/safe motherhood 1996 WHO press, Geneva

[B10] World Health Organization Integrated management of pregnancy and childbirth. WHO recommended interventions for improving maternal and newborn health 2009 WHO press, Geneva

[B11] Scott KD Berkowitz G Klaus M A comparison of intermittent and continuous support during labor: a meta-analysis Am J Obstet Gynecol 1999 180 5 1054 1059 10329855 10.1016/s0002-9378(99)70594-6

[B12] Zadoroznyj M Social class, social selves and social control in childbirth Sociol Health Illn 1999 21 3 267 289

[B13] Larkin P Begley CM Devane D Women’s experience of labour and birth: an evolutionary concept analysis Midwifery 2009 25 e49 e59 17996342 10.1016/j.midw.2007.07.010

[B14] Pascali-Bonaro D Kroeger M Continuous female companionship during childbirth: A crucial resource in times of stress or calm JMWH 2004 49 suppl 1 19 27 15236700 10.1016/j.jmwh.2004.04.017

[B15] Meyer BA Arnold JA Pascali-Bonaro D Social Support by Doulas During Labor and the Early Postpartum Period Hospital Physician 2001 37 9 57 65

[B16] Ministry of Health (Nepal), New ERA, ORC Macro Nepal Demographic and Health Survey 2006 2007 Family Health Division, Ministry of Health, His Majesty’s Government, Kathmandu, Nepal; New ERA, Kathmandu, Nepal and ORC Macro, Calverton, MD

[B17] Mullany BC Barriers to and attitudes towards promoting husbands’ involvement in maternal health in Kathmandu, Nepal Soc Sci Med 2006 62 2798 2809 16376007 10.1016/j.socscimed.2005.11.013

[B18] Sapkota S Kobayashi T Takase M Husbands’ experiences of supporting their wives at childbirth in Nepal Midwiferyin press 10.1016/j.midw.2010.10.010 21129829

[B19] Sreeramareddy CT Joshi HS Sreekumaran BV Giri S Chuni N Home delivery and newborn care practices among urban women in western Nepal: a questionnaires survey BMC Pregnancy Childbirth 2006 6 27 16928269 10.1186/1471-2393-6-27 PMC1560161

[B20] Central Bureau of Statistics Nepal Living Standard Survey 2003/04. Statistical report, Volume one 2004 Central Bureau of Statistics, National Planning Commission, His Majesty’s, Government of Nepal

[B21] Caltabiano M Castiglioni M Changing family formation in Nepal: Marriage, cohabitation and first sexual intercourse Int Fam Plan Perspect 2008 34 30 39 18440915 10.1363/ifpp.34.030.08

[B22] Sapkota S Kobayashi T Takase M Women’s experience of giving birth with their husband’s support in Nepal BJM 2011 19 7 426 432

[B23] Vaidya S KC I Shrestha P Statistics of Paropakar Maternity and Women’s Hospital 2066/2067 2010 Smarika: 51th Anniversary.Bagmati offset press, Kathmandu 59 62

[B24] Ho R Handbook of univariate and multivariate data analysis and interpretation with SPSS 2006 Chapman and Hall/CRC, USA

[B25] Hodnett ED Lowe NK Hannah ME Willan AR Stevens B Weston JA Ohlsson A Gafni A Muir HA Myhr TL Stremler R Effectiveness of nurses as providers of birth labor support in North American hospitals JAMA 2002 288 11 1373 1381 12234231 10.1001/jama.288.11.1373

[B26] Hodnett ED Pain and women's satisfaction with the experience of childbirth: a systematic review Am J Obstet Gynecol 2002 186 5 Suppl Nature S160 72 12011880 10.1067/mob.2002.121141

[B27] Pirdel M Pirdel L Perceived environmental stressors and pain perception during labor among primiparous and multiparaous women J Reprod Infertil 2009 10 3 217 23 PMC3719331 23926472

[B28] Hoelter LF Axinn WG Ghimire DJ Social change, premarital nonfamily experiences and marital dynamics J Marriage Fam 2004 66 1131 1151

[B29] Mullany BC Becker S Hindin MJ The impact of including husbands in antenatal health education services on maternal health practices in urban Nepal: results from a randomized controlled trial Health Educ Res 2007 22 166 176 16855015 10.1093/her/cyl060

[B30] Christiaens W Verhaeghe M Bracke P Pain acceptance and personal control in pain relief in two maternity care models: a cross-national comparison of Belgium and the Netherlands BMC Health Serv Res 2010 10 268 279 20831798 10.1186/1472-6963-10-268 PMC2944275

[B31] McCrea H Wright ME Stringer M Psychosocial factors influencing personal control in pain relief IJNS 2000 37 493 503 10.1016/s0020-7489(00)00029-8 10871659

[B32] Nunnally JC Psychometric theory 1978 2 McGraw- Hill, New York

